# Functional Characterization of Human Pluripotent Stem Cell-Derived Models of the Brain with Microelectrode Arrays

**DOI:** 10.3390/cells11010106

**Published:** 2021-12-29

**Authors:** Anssi Pelkonen, Cristiana Pistono, Pamela Klecki, Mireia Gómez-Budia, Antonios Dougalis, Henna Konttinen, Iveta Stanová, Ilkka Fagerlund, Ville Leinonen, Paula Korhonen, Tarja Malm

**Affiliations:** 1 A.I. Virtanen Institute for Molecular Sciences, Faculty of Health Sciences, University of Eastern Finland, 70211 Kuopio, Finland; pistono.cristiana@gmail.com (C.P.); pamela.klecki@hotmail.com (P.K.); mireia.gomez.budia@uef.fi (M.G.-B.); antonios.dougalis@uef.fi (A.D.); henna.konttinen@uef.fi (H.K.); iveta.stanova@uef.fi (I.S.); ilkka.fagerlund@uef.fi (I.F.); paula.korhonen@uef.fi (P.K.); tarja.malm@uef.fi (T.M.); 2 Department of Neurosurgery, Kuopio University Hospital, 70029 Kuopio, Finland; ville.leinonen@kuh.fi; 3 Neurosurgery, Institute of Clinical Medicine, Faculty of Health Sciences, University of Eastern Finland, 70029 Kuopio, Finland

**Keywords:** pluripotent stem cells, disease modeling, differentiation, functional characterization, microelectrode array, neuronal network, cell phenotype, neurons, brain

## Abstract

Human pluripotent stem cell (hPSC)-derived neuron cultures have emerged as models of electrical activity in the human brain. Microelectrode arrays (MEAs) measure changes in the extracellular electric potential of cell cultures or tissues and enable the recording of neuronal network activity. MEAs have been applied to both human subjects and hPSC-derived brain models. Here, we review the literature on the functional characterization of hPSC-derived two- and three-dimensional brain models with MEAs and examine their network function in physiological and pathological contexts. We also summarize MEA results from the human brain and compare them to the literature on MEA recordings of hPSC-derived brain models. MEA recordings have shown network activity in two-dimensional hPSC-derived brain models that is comparable to the human brain and revealed pathology-associated changes in disease models. Three-dimensional hPSC-derived models such as brain organoids possess a more relevant microenvironment, tissue architecture and potential for modeling the network activity with more complexity than two-dimensional models. hPSC-derived brain models recapitulate many aspects of network function in the human brain and provide valid disease models, but certain advancements in differentiation methods, bioengineering and available MEA technology are needed for these approaches to reach their full potential.

## 1. Introduction

Human pluripotent stem cells (hPSCs) have the capacity to differentiate into any cell type of the body [1], which has enabled the creation of in vitro models of cells and tissues of human origin. The first hPSC-derived electrically active neurons were differentiated from human embryonic stem cells (hESCs) two decades ago [2]. However, hESCs were available mainly to institutions and research groups with access to unused embryos from fertility clinics, and their use suffered from ethical issues [3]. This changed in 2007 when Takahashi et al. published a method for reprogramming human adult fibroblasts back into stem cells using retroviral overexpression of four specific transcription factors (Octamer-binding transcription factor 4 (Oct4), SRY-box transcription factor 2 (Sox2), Kruppel like factor 4 (Klf4), and the MYC proto-oncogene, bHLH transcription factor (c-Myc)) [4]. This not only widened the availability of hPSCs and reduced the associated ethical burden, but significantly facilitated the development of personalized medicine as the human-induced pluripotent stem cells (hiPSCs) could be differentiated into patient-specific in vitro disease models of practically any tissue or organ, including the brain [5]. Lately, research has been focused on the self-organizing three-dimensional (3D) hPSC-derived brain models known as organoids [6], even though two-dimensional (2D) cultures of human neural cells continue to serve as functional models of the brain [7].

**Figure 1:**
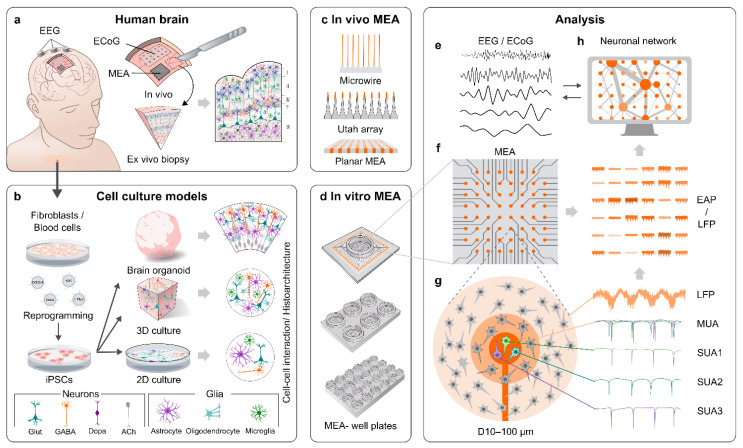
MEA recordings of the human brain and corresponding hPSC-derived models. (**a**) Neuronal network activity can be recorded directly through the skull and skin using EEG or on the surface of the brain using ECoG or MEA. It is possible to attain ex vivo biopsies of the living human brain during brain surgeries for electrophysiological measurements. (**b**) Another option to gain access to human neuronal networks is to first reprogram human somatic cells into hiPSCs, which are then differentiated into different cell types of the brain (Glut = glutamate, GABA = gamma-aminobutyric acid, Dopa = dopamine, Ach = acetylcholine). Current methods enable differentiation of the iPSCs into 2D, 3D, or even self-organizing organoid models of the brain. (**c**) MEA formats, such as microwires and Utah arrays, have been used to record the activity of the human brain in vivo. (**d**) Various MEA systems for recording in vitro cell cultures are also commercially available. (**e**) The network data provided by EEG and ECoG are filtered similarly as the local field potential (LFP) data from MEA. (**f,g**) Electrode data from MEAs can be filtered for LFPs or extracellular action potentials (EAPs). (**g**) A single electrode can detect EAPs from multiple neurons in its vicinity. The resulting data are referred to as multi-unit activity (MUA), but if the EAPs from different neurons are discriminated based on the waveform shape, the resulting data are referred to as single-unit activity (SUA). (**h**) Simultaneous measurement of neuronal activity from multiple electrodes at different locations across the sample on MEA allows the analysis of the functional connectivity in the neuronal network.

Microelectrode arrays (MEAs; also referred to as multielectrode arrays) are a tool for the functional characterization of electrically active cell populations, including neurons (Figure 1a,c–g). MEA recordings have been performed on the human brain in vivo for decades [8,9,10,11,12] and, thus, there is a distinct reference point in the literature on how MEA data from hPSC-derived brain models should ideally look. Certain elements of MEA data can also be directly compared to electroencephalography (EEG) data [13]. However, comparisons are complicated by the fact that hPSC-derived models tend to represent early developmental stages [13] and practically all in vivo MEA data originate from adult individuals [8,11,12], which can require animal models to fill in the gaps [14]. MEAs are a non-invasive method from the perspective of the recorded cells, because, in contrast to intracellular whole-cell clamp-recording techniques, MEAs register potential changes in the extracellular space. MEAs also do not require loading of the target cells with dyes, such as calcium indicators [15]. This enables monitoring of the same target for up to several months [16,17], even though a significant challenge in clinical applications such as brain–machine-interfaces (BMIs) is that the array needs to remain functional and safe for several years, preferably even decades [18,19]. Another advantage of MEAs is the simultaneous monitoring of the tissue or culture with multiple electrodes, which in neuroscience enables the measurement of neuronal network activity and connectivity [20,21]. MEAs can, therefore, provide a view into the network activity of the human brain and its corresponding hPSC-derived models.

However, is it necessary to be species-specific and use hPSC-derived models (Figure 1b) when describing the human brain? The work by Hodge et al. showed major differences between humans and mice in the proportions, laminar distributions, gene expression and morphology of cortical cells, as well as in the serotonin responsiveness of specific circuits [22]. The dendrites of human pyramidal neurons in the cortex are also less excitable when compared to rat dendrites due to lower ion channel densities, which results in reduced burst firing and increased compartmentalization in signal transduction in humans [23]. Neuronal activity may also be coupled to different neural oscillations in the brains of humans and rodents [24]. Species differences are not limited to neurons. Human astrocytes are larger, more complex and diverse [25,26], and display greater susceptibility to oxidative stress as well as reduced capacity for neuronal repair and increased antigen presenting properties after hypoxia in comparison to their rodent counterparts [27]. Human microglia exhibit differences in subpopulations and transcriptional states in comparison to rodent microglia [28,29]. In drug development, the various species differences have manifested as repeated failures of putative Alzheimer’s disease (AD) therapies after animal testing [30], and as a rule of thumb, only 6% of putative central nervous system (CNS) drugs achieve clinical approval [31]. While animal models have provided (and will continue to provide) valuable results and insights in neuroscience, relevant hPSC-derived models are needed to complete the picture of human brain development and maturation in order to aid evidence-based drug discovery [14].

In this review, we summarize the latest research on the functional characterization of hPSC-derived neural networks using MEA and determine the aspects in which the data can be compared to the human brain. We first discuss the different types of MEAs used for in vivo and in vitro recordings. Next, we examine the literature on how the electrical activity of the human brain looks in MEA recordings. We thereafter summarize 2D hPSC-derived neural cultures on MEA, their strengths, weaknesses, and what kind of information they can provide us on the physiology and pathology of neuronal networks. As organoids and other hPSC-derived 3D models have already been in use for several years, we take a similar look into their MEA data, their ins, outs, and how they can describe the structure and function of the human brain. We finalize the review by hypothesizing how the functional characterization of hPSC-derived brain models with MEAs could be developed in the foreseeable future.

## 2. MEAs

### 2.1. General Properties of MEAs

MEAs consist of small electrodes arranged in a predetermined grid on a rigid or flexible base material. Advances in materials for electrode fabrication, insulation methods and electronic design have enabled the low-cost production of MEAs with highly dynamic properties and low impedances that can both acquire continuous extracellular recordings and deliver electrical stimulation to the tissue or culture in a predefined fashion [32,33]. The typical electrode diameters seen in MEAs range from tens of micrometers to a few micrometers, which roughly correspond to individual neuron somas and even smaller structures (neurites). The electrodes enable the registering of an electrical signal containing both extracellular action potentials (EAPs, also known as “spikes”) occurring as a result of firing of single cells, and local field potentials (LFPs), which represent synaptic current summation (input) to a given area [34,35]. The EAPs are detected from the high-frequency band-pass-filtered part of the signal (typically 300–3000 Hz) and the LFPs from the low-frequency band-pass-filtered part (typically 1–200 Hz) (Figure 1f–g). MEAs enable the detection of neural oscillations (brain waves) from the low-frequency part of the signal, similar to electrocorticography (ECoG) and EEG [13,24,35] (Figure 1a,e,g), and allow the monitoring of fundamental operational properties of both the developing and mature network of neurons, thus giving the neuroscientist an unprecedented view into the spatial and temporal dynamics of a brain.

### 2.2. MEAs for In Vivo Applications

In vivo MEA measurements refer to applications in which electrodes are implanted directly into the brain (Figure 1a,c). Modern MEAs for in vivo applications have greatly evolved from wire electrodes such as the one used by Strumwasser in the 1950s to record squirrel neurons [36]. Microwire tetrodes and other microwire setups have since continued to provide data from animal models [37,38,39] and also from deep-brain structures of humans [9]. The first array with surface embedded electrodes, known as the Michigan array (NeuroNexus), consists of planar titanium/iridium electrodes on a silicon-based shank [40,41]. The Michigan array was also modified for 3D electrode configurations [42]. The incorporation of complementary metal-oxide-semiconductor (CMOS) technology into the arrays [43] enabled sub-cellular resolution through smaller electrode sizes and pitches [44]. The CMOS technology was later up-scaled in the Neuropixels array, which has 960 12 × 12 μm electrodes on a silicon shank arranged to a four-column checkerboard grid [45]. Another famous silicon-based array, the Utah array (Blackrock Microsystems), consists of 96 1.5 mm high silicon needles with 400 μm pitch, arranged in a 10 × 10 grid [46]. The sharpened tip of each electrode is coated with gold/platinum, and the rest of the array is insulated with polyimide. Laminar probes, such as the U-probe (Plexon), are designed for mapping the activity of different cortical layers with primarily linear layouts of planar electrodes [47]. Electroactive neurotransmitters such as dopamine can be detected in real-time using voltammetric methods and planar platinum electrodes [48]. Non-electroactive neurotransmitters such as glutamate can also be detected when the electrodes are coated with specific enzymes [49]. Various array designs with flexible base materials such as polyimide or parylene-C have also been created [50,51,52,53,54] and they can even be 3D-printed [55]. However, apart from a recent study on flexible 2D arrays [53], practically all currently available MEA data from the human brain were obtained with a few one-dimensional arrays such as laminar probes [12], 2D Utah-type arrays [11,56], or microwires [8,9,57].

### 2.3. MEAs for In Vitro Applications

The first MEAs for in vitro recordings were developed in the 1970s [58]. The most wide-spread arrays are the 8 × 8 grids of planar titanium nitride (Multi Channel Systems) or indium tin oxide electrodes (MED64) at the bottom of a cell culture dish. The electrodes of standard MEAs are typically embedded in a thin glass base, which facilitates real time microscopy (e.g., calcium imaging) of the tissues or cultures around the electrodes [59], even though the electrodes themselves could also be made transparent [60,61]. CMOS technology has also been incorporated into in vitro MEAs, enabling the construction of very high-density arrays for measurements from different parts of the same cell and the analysis of properties such as signal propagation velocity within individual cells [62,63]. Multi-well MEAs (Figure 1d), such as the Maestro system (Axion Biosystems), have made high-throughput MEA screening possible [64]. Neurotransmitter-detecting arrays were also developed for in vitro applications [65]. Many research groups have the capability to produce custom-made arrays, which combined with microfluidic devices, enable the study of controlled artificial networks [66,67,68]. While most in vitro MEAs have planar electrodes, arrays with sample-penetrating pyramid-shaped electrodes are commercially available, especially for tissue slice experiments [69]. However, true 3D in vitro arrays (recorded simultaneously using multiple 2D planes) were also recently developed and incorporated with 3D neural cultures [70]. In summary, the technological developments in the in vivo MEAs have also been translated to in vitro applications and there is very little in terms of MEA technology hindering the modeling of the human brain with hPSC-derived neural cultures.

## 3. MEA Recordings of the Human Brain

### 3.1. Requirements for MEA Recordings Set by In Vivo Neuronal Properties

The measurement of neuronal electrical activity with MEAs requires the overcoming of several technical challenges, which is exacerbated in EAP recordings and related data analysis. In order to detect EAPs from individual neurons, electrode diameters below 50 μm are desirable, and smaller electrode sizes (and densities) bring higher spatial resolution [71]. However, as electrode size is inversely related to electrode impedance, and thus, to electrode noise, this would also pose a limit to the events that can be successfully discriminated by electrodes, as well as to how much current can be injected from a given electrode when it is used for stimulation. A signal conduction velocity of 50–60 m/s in myelinated human neurons [72] and the ≤ 1.5 ms EAP duration [21,59] necessitate signal acquisition rates as high as 10 or even 50 kHz [71]. EAPs are typically detected when their amplitude exceeds a certain threshold, which is often set to 4–6 times the standard deviation of background noise, and the detections can be verified with principal component analysis [73] or by comparing the spike shape to a template (wavelet) [74,75]. Signal detection is complicated by the low amplitude of EAPs (~20–100 μV) [39,76], which is affected by electrode resistance and decreases with increasing neuron–electrode distance according to an inverse-square rule [51]. The low signal amplitudes require a combination of amplification and recording systems with sufficiently high signal-to-noise ratios [71]. However, a Ø 12.5 μm brain-implanted microelectrode can still receive signals from at least a 50 μm radius [38] and because the human cortex, for example, contains 44,000 neurons per mm^3^ [77], a single electrode can potentially receive EAPs from tens, or even hundreds of neurons [39]. These EAP data are often referred to as multi-unit activity (MUA). The MUA data can be further processed with algorithms for spike discrimination by waveform shape [74,78]. Based on the waveform shape, the EAPs can be assigned to a single neuron that is firing, and the resulting data are often referred to as single-unit activity (SUA).

### 3.2. Challenges Set by the 3D Structure of the Brain to MEA Recordings

The spatial organization of the human brain also sets challenges to the measurement technology and to the models that describe the brain. The human cerebral cortex is 2–3 mm thick on average and divided into six layers, each comprising specific neuronal types [22] and electrical activity [12,79,80]. The cortex is also divided into cortical columns, where pyramidal cells mainly orientate vertically but interneuron projections also spread horizontally, creating a 3D structure [81]. Due to its location, the cortex is the easiest brain structure to sample with MEAs in epilepsy studies and with BMIs [12,82,83,84,85]. Accordingly, most available MEA data from the in vivo human brain originate from the cortex and the most direct comparisons in terms of MEA data can be drawn between the cortex and its in vitro models. Still, in some reports, depth electrodes and microwires have successfully mapped the activity of neurons in deeper brain structures below the cortex, e.g., in the hippocampus [76,86,87]. Long neuronal pathways, on the other hand, are outside the reach of typical arrays due to their sheer size, except when probed with multiple arrays [87]. Though the 3D structure of the brain limits direct observations of neuronal activity from deep brain structures, the 3D neural anatomy and neuronal circuitry are also important features that models should ideally replicate.

### 3.3. Non-Neuronal Cells Affect Neuronal Activity

The brain also contains non-neuronal cell types and tissues. However, due to the invasiveness and complexity of necessary research methods, their effects on MEA data have been mainly evaluated in animal models. The data from these animal studies showed that oligodendrocytes, by wrapping axons with myelin, increase neuronal signal conduction velocity and promote the development of neuronal network synchrony [88]. Astrocytes form their own networks and increase the activity and synchronization of neuronal networks [89], which is important to, e.g., hippocampal-prefrontal synchronization and cognitive function [90]. Activation of microglia, the immune cells of the brain, causes microglia to remove inhibitory presynaptic terminals and, thus, increase LFP activity in the 20–40 Hz range (high beta-low gamma) [91]. Increased supply of oxygen, glucose and nutrients through the blood vessels of the brain (a hemodynamic response) is associated with steep increases in MUA and LFP counts [92,93]. There are virtually no data on the impact of specific non-neuronal cells on neuronal network function in the human brain. However, the development of hPSC-derived brain models containing glial cells and vasculature presents an opportunity to study the effect of non-neuronal cells on network activity in a human context [73,94].

### 3.4. Neuronal Firing in the Human Brain in MEA Recordings In Vivo

How does human neuronal activity then look in MEA recordings in vivo? Cortical mean firing rates of EAPs (SUA) from 0.1 to 1.4 Hz h are typical in the literature [53,95,96,97], even though values from 4 to 7 Hz have also been reported [76,98]. The differences in mean firing rates between studies most likely reflect differences in spike detection [74] and sorting methods [78], although other factors such as exact measurement positions affect this as well. Arousal can also affect the activity. The hippocampal mean firing rate (SUA) was shown to drop from 2 Hz during wakefulness to 1.2 Hz in rapid eye movement (REM) sleep [99], while the cortical mean firing rate was found to be reduced by more than 80% in propofol anesthesia [100]. However, a 2016 study found no significant differences in cortical firing rates between wake and sleep states [101].

Different neurons also display different activity patterns [37]. Peyrache et al. studied layer II/III middle temporal gyrus neurons during sleep with a Utah-type array (Neuroport, Blackrock Microsystems) and discriminated the detected MUA to pyramidal cells and interneurons according to the amplitude and duration ratios of the negative and positive phases of the EAPs [21]. The resulting mean firing rates (SUA) were approximately 0.3 Hz for the pyramidal cells and 2 Hz for interneurons, and very similar results were also attained in another recent study [102]. However, a 2016 study of the same region suggested mean firing rates (SUA) of 1.9–2.6 Hz for pyramidal cells and 6.4–8.1 Hz for interneurons, with slight, but statistically non-significant variation resulting from sleep–wake states [101]. Overall, 24% of neurons detected by Peyrache et al. were deemed as interneurons [21], concurring with the generally accepted 20–30 % portion of interneurons in the cortex [81].

In the work by Peyrache and co-workers, inter-spike interval (ISI) analysis verified that the identified interneurons fired EAPs in a train-like pattern, while the pyramidal cells also exhibited bursts of EAPs [21]. Other studies have examined burst rates and found that the mean burst rate (SUA) in the cortex is approximately seven per min during wakefulness and 5–3.1 during sleep [95,98]. Hippocampal mean burst rates (SUA) are between 3 and 5 per min during wakefulness and slow-wave sleep (SWS) but drop closer to 1 per min in REM sleep [98]. Taken together, variability between studies makes it difficult to determine what the mean firing rate in hPSC-derived models should be. Still, brain models should contain both interneurons (mostly GABAergic) and pyramidal cells (glutamatergic) and display steadily repetitive firing as well as bursts under basal and induced conditions.

### 3.5. Neural Oscillations of the Human Brain in MEA Recordings In Vivo

In addition to EAPs, neural oscillations can also be detected with MEAs. However, what are the main documented oscillation characteristics that should be replicated in hPSC-derived models? Jacobs et al. studied LFPs in addition to SUA and found that the probability of detecting SUA correlates with different neural oscillations depending on the brain region: frontal cortex SUA correlated with gamma waves, temporal and parietal cortex SUA with theta waves, hippocampal SUA with delta and gamma waves, parahippocampal SUA with gamma waves and amygdala SUA with delta, beta and gamma waves [24]. During sleep and anesthesia, cortical neuron EAP occurrence mainly correlates with delta and slow waves [95,100], even though gamma-wave-correlating SUA during SWS has also been found [87,101]. However, when LFP activity alone was studied with MEA, it was found that the dominant activity in the cortex during both wakefulness and sleep was the theta and delta band activity of the superficial cortical layers (I–II) [80]. In summary, EAPs in conjunction with neural oscillations are an important functional feature that brain models of MEAs should replicate, and finding delta and gamma waves in cortical models is of particular interest.

### 3.6. Functional Connectivity of the Human Brain in MEA Recordings In Vivo

One of the main advantages of MEAs is the ability to measure functional connectivity as the temporal or spectral correlation of signals (Figure 1e–f), but how is this displayed in recordings from the human brain? Given their size, individual MEAs can study only millimeter-scale or smaller areas [57,71] and large-scale connectivity is better studied with ECoG, EEG or functional magnetic resonance imaging (fMRI). Still, Le Van Quyen et al. [87] implanted 8–14 depth electrodes with microwires in different sites around the cortex in nine patients and discovered MUA during SWS that occasionally occurred simultaneously across all recording sites along gamma waves. This indicates transient large scale functional connectivity during SWS, and other studies have found that functional connectivity within a single MEA is highest during SWS [98,101]. A combined ECoG/MEA study demonstrated that while propofol-induced anesthesia disrupted most large-scale functional connectivity between cortical areas > 2 cm apart, SUA connectivity largely remained within a NeuroPort (Blackrock Microsystems) MEA (<4 mm distance) implanted in layer II/III of the temporal gyrus [100]. LFP connectivity in the same area was later found to heavily depend on the electrode-to-electrode distance (strongest ≤ 3 mm) [101]. Functional connectivity of the region was also found to follow the crude overall orientation of pyramidal cells and interneurons in the cortex [81]; MEA-wide connectivity between interneurons remained irrespective of distance, whereas the connectivity between pyramidal cells decreased with increasing cell-to-cell distance [21]. Taken together, brain models of MEAs should display array-wide functional connectivity and cortical models should ideally replicate the functional connections of cortical columns and layers.

### 3.7. Detecting Epileptic and Other Pathological Activity in the Human Brain with MEAs In Vivo

The majority of available MEA data from the human brain are attained through array implantations that are justified by medical procedures related to epilepsy [21,87,96,98]. This may skew our knowledge of brains unaffected by seizures, even though some data also originate from BMIs implanted in spinal cord injury patients [11,56]. It is also noteworthy that there is a distinct lack of human MEA data from other disorders affecting brain function, such as AD, Parkinson’s disease (PD) or stroke, which underlines the need for creating appropriate in vitro models of such disorders. Still, the measurements from epilepsy patients provide an excellent reference for in vitro models of epilepsy on MEAs.

It has been found that long-lasting, ictal electroencephalic seizures are not the only defining neurophysiological characteristic of human epilepsy. MEAs can also detect interictal high-frequency events in epilepsy patients, but their role in epileptogenesis is still not clear [12,103,104,105]. However, epilepsy is not a single disease but a group of disorders and seizure types vary accordingly: during a seizure, the ictal core can experience clear increases in MUA [106], no increases whatsoever [107], or particular neurons can be silenced altogether [85]. Even though general synchrony and mean firing and burst rates are elevated in epileptic brain areas [98], only certain seizure types, particularly those associated with spike–wave complexes, exhibit stereotypical rhythmic synchronous bursting [84]. A more uniform finding in MEA recordings is a 0.5–3 min post-ictal quiescence of SUA/MUA in the ictal core [84,85,106]. Furthermore, MEA recordings have shown that the mechanisms of epilepsy are not local but both ictal and interictal events are affected (and predicted) by the activity of distant neurons [12,107,108,109]. hPSC-derived epilepsy models of MEAs should, therefore, take functional connectivity and complex neuronal networks into account. Due to the variety of seizure types, it can be challenging to identify seizure-like activity in vitro, but controlled experiments should detect ictal and interictal events in networks engineered to be epileptic.

### 3.8. Detecting Activity of the Human Brain with MEAs Ex Vivo

An important sample type for bridging the gap between the human brain and in vitro models are the cortical and hippocampal ex vivo slices obtained from epilepsy surgeries. However, the samples are rare and delicate, and can require chemical stimulation (low Mg2^+^, high K^+^ or 4-aminopyridine) to display ictal or interictal activity [82,110,111]. Still, it is possible to see spontaneous network and epileptic activity in human slices [82,112]. The slice lifetime can be increased to several weeks by maintaining them as organotypic cultures in human cerebrospinal fluid (CSF) [113]. CSF can also increase synchronous bursts and excitability in an ex vivo slice in less than one hour [114]. Importantly, electrophysiological recordings of slices showed that the phenomena detected in the intact brain can also be detected with in vitro methods [112].

**Figure 2:**
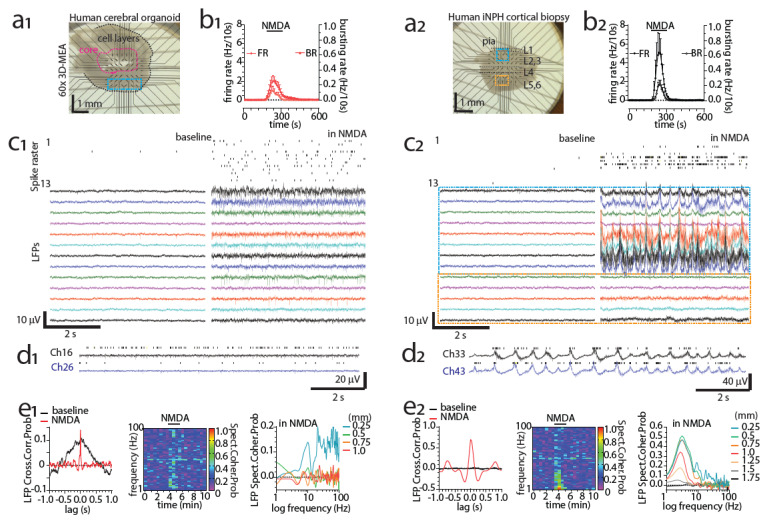
Functional interrogation of human cerebral organoids and acutely excised iNPH cortical slices using MEA. (**a_1,_ a_2_**) Image of a cerebral organoid slice (**a_1_**) and an iNPH patient cortical slice (**a_2_**, frontal lobe biopsy) recorded with a 3D-MEA device hosting 60 pyramidal-tip-shaped titanium nitride (TiN) electrodes (100 µm in height), spaced at 250 µm and insulated by a thin layer of silicon nitride (Multichannel Systems). Blue/orange areas denote areas with channels of interest/activity, black dotted margins denote approximate cortical layers or cell layers, and magenta lines denote approximate borders of organoid core. (**b_1_**,**b_2_**) Spike firing and spike bursting rate timelines upon a brief exposure (2 min) to N-methyl-D-aspartate (NMDA) bath superfusion (200 µM) for a cerebral organoid slice (**b_1_**) and an iNPH patient cortical slice (**b_2_**). Bursting was defined as at least three spikes occurring above 6 standard deviations in the smoothed firing histogram (0.1 s bins, 3 widths smoothing routine). (**c_1_**,**c_2_**) Representative spike raster activity (top, resulting from 300 to 3000Hz band-pass filtering of the raw signal followed by a 5.5 standard deviation thresholding for spike detection) and raw local field potentials (LFP, 1–200 Hz band pass filtering of raw signal) recorded at baseline and in the presence of 200 µM NMDA from a cerebral organoid (13 channels, taken from the blue area shown in **a_1_**) and iNPH cortical slice (13 channels, taken from blue and orange areas shown in **a_2_**). (**d_1_**,**d_2_**) A 10s raw data detail of NMDA-induced activity (spikes and LFP) at two adjacent (250 µm) representative channels located at a vertical depth of ~400–500 and 600–700 µm from border/pial surface for a cerebral organoid and an iNPH cortical slice, respectively. Note the strong delta (δ) band (0.5–3 Hz) LFP synchronization occurring in the iNPH slice and the time locked LFP-spike sequences. (**e_1_**,**e_2_**) Comparative, time and frequency domain analysis of markers of connectivity for NMDA-induced effects in LFPs for the pairs of channels presented in **d_1_,d_2_**. Cross-correlation probability analysis of time series (left), cross-spectral coherence probability timeline (mid) and baseline-subtracted spectral coherence probability against channel vertical distance, collectively demonstrating the relatively weak, spatially and spectrally restricted, NMDA-induced synchronicity for the organoid circuit against the robust behavior of the human cortical slice.

Epilepsy surgeries are not the only possible source of human brain samples for MEA recordings. In Figure 2(a_2_), we present another possibility, an acute cortical ex vivo slice obtained from an idiopathic normal pressure hydrocephalus (iNPH) patient during shunt implantation [115,116]. The shunt reduces intracranial pressure by draining the excess CSF into the abdominal cavity of the patient, and the millimeter-scale diagnostic cortical biopsy was obtained from the shunt implantation site using minimally invasive methods. The spontaneous MEA activity in the slice was minimal, but NMDA (200 μM) was able to induce spiking, bursting (MUA; Figure 2(b_2_,d_2_), delta oscillations Figure 2(c_2_,d_2_,e_2_) and signs of functional connectivity (Figure 2(e_2_) in the sample. In comparison, an acute slice obtained from a hiPSC-derived cerebral organoid (Figure 2(a_1_); differentiation presented in [117]) also displayed NMDA-induced spiking and bursting (MUA; Figure 2(b_1,_c_1_) but displayed no delta-band activity (Figure 2(c_1_,d_1,_e_1_). The spiking and bursting responses in the organoid (Figure 2(b_1_,c_1_) were also lower, and functional connectivity was much more spatially limited (Figure 2(e_1_) in comparison to the human ex vivo slice (Figure 2(b_2,_d_2_,e_2_). In the following sections, we examine how hPSC-derived models of MEAs can be improved to better represent the human brain. We discuss the MEA activity of hPSC-derived brain models in the literature and which aspects of the data are comparable to data from the human brain.

## 4. hPSC-Derived 2D Brain Models of MEAs

### 4.1. General Properties of 2D Cultures on MEAs and Early hPSC-Derived Brain Models

Two dimensional (2D) in vitro cultures of neural cells have been a robust and relatively low-cost tool in neuroscience for more than 100 years and they are currently commonly used for studying fundamental cell biology as well as to perform toxicological and pharmacological screenings [118]. Two-dimensional MEA models are relatively simple-to-use, reproducible and provide homogenous cultures that are easy to image [59]. The first successful MEA recordings of hESC-derived 2D neuronal models were performed more than a decade ago by Heikkilä et al. [119]. Perhaps surprisingly, these first 2D models were based on neurons pre-differentiated in 3D as free-floating neurospheres [119,120]. The networks showed spontaneous firing, responses to pharmacological treatment, and synchronous bursts after one month of culturing [119], and were functionally similar to neuronal networks derived from mouse embryonic stem cells (mESCs) [121]. The abundance and variety of bursts detected by Heikkilä et al. in hESC-derived neuronal networks also resembled those observed in developing dissociated rat cortical neurons [122]. The differentiation protocol was later successfully applied to hiPSCs, which also produced electrically active neurons on MEA [123].

Later on, Amin et al. showed that hiPSC-derived neuronal networks can be cultivated on CMOS-MEAs and stimulated electrically [124]. Their work also highlighted the importance of platform-specific coatings in 2D MEA models; neuronal networks grown on poly-dl-ornithine (PDLO)-coated CMOS-MEA showed increased firing, enhanced synapse maturation and reliable responses to electrical stimuli in comparison to polyethylenimine (PEI) coating, which is often favored in standard MEAs. However, these pre-coatings need to be supported by a final coating using a part of the extracellular matrix (ECM), usually laminin. Human recombinant laminins, especially those contained in the α5-chain, are optimal for hPSC-derived neuronal networks on MEA [125]. The early 2D human neuron cultures on MEA established methodological ground rules such as proper substrate coatings, which showed the potential of the approach in studying neuronal function.

### 4.2. Human Neurons Produced with Dual-SMAD Inhibition Differentiation on MEAs

Many current neuronal 2D differentiation methods utilize two essential steps: (1) the induction of neuronal differentiation and (2) the proliferation of the induced cells while neuronal connections are created [126,127]. The induction phase is often achieved with small-molecule inhibitors of the activin/nodal and bone morphogenic protein (BMP) pathways (so-called dual-SMAD inhibition), while the neuronal proliferation is typically induced with basic fibroblast growth factor (FGF2). In 2019, Hyvärinen et al. showed that such differentiation can produce cortical-like human neurons that display firing (MUA) at 4.4 Hz, burst rates of up to 33 bursts per min and array-wide functional connectivity after 46–56 days of differentiation [128]. The burst rate appeared rather high but depended heavily on the maturation stage. However, another study reported burst rates of the same protocol of between 2.5 and 3 per min [129], which is more in line with the in vivo data [95,98], even though a direct comparison is unreliable due to the in vivo data being expressed as SUA and the in vitro data as MUA. Immunohistochemistry and pharmacological manipulations showed the presence of functional glutamatergic and GABAergic neurons in the culture [128]. Astrocytes were also clearly present. Shimba et al. achieved similar cell identities and high MEA activity with dual-SMAD inhibition, even though they also reported 1.3% of neurons in the culture being cholinergic, with 6.7% of neurons being GABAergic and the rest assumed to be glutamatergic [17]. The GABAergic portion is perhaps low considering the 20–30% portion generally reported in the human cortex [21,81]. Even so, the differentiation based on dual-SMAD inhibition appears as a robust method for producing functional human 2D networks containing glutamatergic and GABAergic neurons as well as astrocytes.

### 4.3. Cell-Type Specific Differentiation Methods for Producing 2D Brain Models of MEAs

There are also alternative protocols that can produce a homogenous population of mature neurons with network activity in only a couple of weeks. Pang et al. and Zhang et al. proposed an approach for the differentiation of hiPSC into upper layer cortical neurons in one step by using lentiviral overexpression of the mouse transcription factor neurogenin-2 (Ngn2) [130,131]. In 2017, Frega et al. modified the protocol and created their own hiPSC line with stable Ngn2 overexpression, and were able to grow the neurons on MEAs in co-culture with rat astrocytes [132]. The networks showed mature neuronal morphology, mean MUA firing rates of 2.5 Hz, burst rates of 4 per min and synchronicity in bursting indicating array-wide functional connectivity after only 23 days of culture. The network was reported to be > 95% homogenous of mitogen activated protein-2 (MAP2) positive neurons that received only excitatory post-synaptic potentials in patch-clamp experiments, suggesting them to be almost entirely glutamatergic.

A similar protocol utilizing lentiviral overexpression of the mouse transcription factors distal-less homeobox 2 (Dlx2) and achaete-scute family bHLH transcription factor 1 (Ascl1) was used to create pure GABAergic populations [133] that are functional on MEA after only two weeks of maturation [134]. Even Ascl1 overexpression alone was shown to induce GABAergic neurons that, under the influence of MEA, produce mean firing rates (MUA) of approximately 5 Hz and 3–4 synchronized bursts per min [135]. While the bursting can be seen as contradictory to human in vivo data, which suggest that inhibitory neurons rarely burst, it is impossible to say whether the in vivo recordings have truly identified GABAergic populations due to the absence of any molecular analysis [21]. It is also possible that the experimental conditions in the in vitro preparations [135] encourage bursting. Furthermore, the in vitro and in vivo MEA data should be analyzed for SUA to compare the function of the supposed GABAergic neurons [21,135]. The drawbacks of the Ngn2 and Dlx2/Ascl1-based differentiation methods are that they produce very simplistic models with no other cell types and the produced human neurons are typically cultivated on rat astrocytes. Still, the speed, efficacy and cell-line-to-cell-line repeatability of the methods [7] highlight their feasibility in mechanistic and pharmacological studies of human neuronal networks.

Several approaches to the differentiation of hiPSCs into cells with specific properties have been proposed over the years, allowing the formation of different cells of the nervous system, including astrocytes, microglia and several neuronal subpopulations [5,136,137,138,139,140]. Considering all of the above, it is evident that hPSC-derived neuronal 2D-cultures can produce both mixed and homogenous populations of neurons (as well as glia) that recapitulate basic firing and bursting activity, as well as the functional connectivity of human neuronal networks on MEA [7,128,135]. However, it is difficult to compare the exact spike and burst counts to in vivo studies because of the lack of SUA analysis in studies with hPSC-derived cultures. The comparison of burst counts is further complicated by the variety of different burst detection algorithms in the field [141]. In addition, there are few reports on neural oscillations or LFP analysis in human 2D cultures on MEAs [61], even though many publications report oscillatory firing behavior [119,128,132,142,143].

### 4.4. Main Limitations of 2D hPSC-Derived Neuronal Cultures and Overcoming Them

Although 2D cultures of hPSC-derived neurons can produce network function, it is important to consider that they are still insufficient to mimic the complexities of the human brain. Many studies have shown that the microenvironment of the culture is extremely important in the regulation of neurogenesis and survival of hPSC-derived neural cultures [130,144]. In this regard, the cells in 2D models are limited to side-by-side contact, lacking a 3D microenvironment and the relevant interactions between the cells and the surrounding ECM [145]. Indeed, it was shown that differentiating neural stem cells respond to stiffness and elasticity of the surrounding scaffold by yielding different ratios of glia and neurons [146].

The second limitation is the generally low or slow maturation of hPSC-derived cultures. The culture time required for obtaining fully functionally mature neurons and networks from hPSCs is not well known and the criteria for “functional maturity” of an in vitro neuronal network is vague and varies between studies. One sign of functional maturity is the ability of GABA to induce inhibitory responses, as it is an excitatory neurotransmitter in early development [147]. A decrease in excitatory GABA responses correlates with the emergence of synchronous network activity in pre-differentiated neurons on MEA, but the remaining excitatory GABA responses also contribute to the synchronous activity seen during development [148]. To obtain more information about the maturation of human neuronal networks, Odawara et al. cultivated pre-differentiated commercially available hiPSC-derived neurons on MEA for over one year [16]. Reaching the endpoint of electrically evoked responses and modulation of activity by glutamate and GABA receptor ligands required more than 230 days. By this time, the firing (MUA) plateaued at approximately 6 Hz, while the number of synchronized bursts reached six per min. It was also suggested that reaching functional maturity on MEA can take 300 days from the beginning of neural induction [17]. However, a low level of functional maturation is not automatically a drawback if the goal is to model, for example, developmental neurotoxicity and associated activity in the prenatal human brain [149,150].

While the first limitation (3D microenvironment) cannot be fully overcome in a 2D environment, the second one (slow maturation) can perhaps be tackled with viral overexpression of cell-type-specific transcription factors [130,131,132,133]. A third limitation of neuronal 2D cultures is that the networks in 2D are formed in random orientations and not in a regulated manner as in the actual brain. This can be addressed, to an extent, with micro-engineered culture environments, as discussed in the section below.

### 4.5. Guiding the Orientation of 2D Cultures

Directed connectivity in artificial neural circuits can be obtained in vitro by guiding the neurites between isolated groups of cells. This can be achieved with microfluidic technology, developed in the early 1990s [151]. The microfluidic devices are constructed of microchambers connected by microchannels and functionalized microdomains whose dimensions range from micrometers to hundreds of micrometers. Polydimethylsiloxane (PDMS) is the most frequently used material for microfluidic chip fabrication but also other materials such as polycarbonate, polyetherimide, silicon, glass, hyaluronic acid, matrigel, collagen, silk protein or agarose are used [152]. Microfluidic chips can be incorporated with 2D MEAs [153,154,155] and the chambers can be connected by microchannels that are narrow enough to prevent the passage of neuron somas, and long enough (400 µm) to allow the passage of axons but not dendrites [156,157,158]. The direction of the neural processes can be affected by adding a “zig-zag” or a “barbed” design to the microtunnels, which can turn one chamber of neurons into “emitting” neurons and another “receiving” neurons [156,157,158]. A similar hierarchy in neuronal signaling can be created by plating one chamber before the other, allowing axons from only one chamber to fill the microtunnels [159]. Microtunnels have also been used to measure axonal signal propagation velocity [68,160] and chips have been designed to allow disruption of functional connectivity between chambers [66], which shows the versatility of the approach.

Still, the studies above utilized rodent neurons, and little has been undertaken to measure the activity of human neurons with MEA-incorporated microfluidic devices. A 2017 study showed that network-encompassing microfluidic tunnels can be used to orientate and focus human neurons on the electrode area, which significantly increases signal detection probability [161]. On a similar note, Shimba et al. reported that human neurons that aggregate and detach from MEA in long-term culture can be pinned down on MEA using microtunnels [17]. The networks in the microtunnel culture remained active for a staggering 450 days and displayed synchronized bursts after 110 days. Firing (MUA) reached a maximum of ~14 Hz at around 270 days, and the burst count at the same time was ~7 per min. A study published in 2020 reported that pre-differentiated hESC-derived cortical neurons formed functional connections between three chambers separated by axon-permitting microtunnels [142]. MEA recordings showed that the neurons formed chamber- and array-spanning synchronous bursts after 49 days in-chip. The activity reached a maximum in approximately 70 days in-chip when firing (MUA) was at 7.8 Hz and burst counts were five per min. The design of the chip allowed pharmacological manipulation of individual chambers, which facilitates the study of the functional connectivity between axonally connected human neuronal networks. In summary, the potential of MEA-incorporated microfluidic devices has been shown, but the methodology could be more widely applied to study, e.g., signal conduction velocity or inter-network signaling in hPSC-derived brain models.

Micropatterning is another technique used to control multi-phase tissue architecture. Almost all micropatterning techniques have their origins in silicon technologies and the microchip industry [162]. Conventional microcontact printing uses a stamping technique to apply molecules such as poly-lysine, proteins, antibodies, enzymes, DNA and also living organisms in a defined pattern on solid scaffolds [162,163]. PLL-laminin microprinting can create multinodal networks of rodent neurons on MEA [164], and it would be interesting to see if human neurons behave differently in such a setting. Other techniques can also be used in combination with microfluidics and micropatterning to guide axonal growth, such as the application of high-frequency electrical fields [165], the creation of a gradient of neurotrophic or growth factors [166], the modification of extracellular matrices [167] and microtopographic surface modification [168]. Ristola et al. combined microfluidic devices and photopatterned microgrooves with 1 µm resolution to orientate human neuronal networks [169]. This kind of combination may provide an option for orienting neuronal networks to resemble, for example, cortical columns, but it is as-yet unknown if such approaches can be combined with MEAs. Bioprinting could be another option [170], but, to our knowledge, neither bioprinting nor micropatterning methods have yet been applied to create pre-patterned human networks on MEA.

### 4.6. hPSC-Derived 2D Neuronal Cultures on MEAs as Models of Physiology

Some works in the literature have used hPSC-derived neurons and MEA to study basic neural physiology. For example, a 2019 work aimed to mimic sleep-wake states in hiPSC-derived dopaminergic neurons [171]. Serotonin was present in the medium during the 12h wake stages, which repeatedly increased synchronized bursts. A sleep-state was modelled in glutamatergic neurons with electrical 1 Hz (slow-wave) stimulation for 15 min every 75 min. The stimulation reduced firing and synchronized bursts repeatedly during the following 15 min period, which resembles sleep-induced brain activity changes in vivo [95,98]. These results are promising, but a more complex study including, e.g., astrocyte function, might provide further insights into human sleep [172]. hiPSC-derived astrocytes in general increase the synchronous activity of neuronal cultures on MEA [94,173]. However, the effect on synchrony appears to require physical contact, as astrocyte-secreted factors alone cause only transient increases in the neuronal firing, but not in synchrony [129]. The activity of different neuron populations was also compared, and hiPSC-derived dopaminergic neurons displayed lower firing rates, burst rates and signal conduction velocities in comparison to motor neurons on CMOS-MEA [143]. Still, it would be very interesting to see similar comparisons between hPSC-derived glutamatergic and GABAergic neurons, especially after SUA analysis.

### 4.7. hPSC-Derived 2D Neuronal Cultures on MEAs as Models of Pathology

hPSC-derived neuronal cultures have also been used in the context of pathological conditions. hiPSC-derived neurons can, for example, elicit synchronized epileptiform bursts [16] and clinically used anti-epilepsy drugs suppressed the epileptiform activity. Furthermore, studying epilepsy in relation to functional connectivity and complex neuronal networks can be facilitated by compartmentalizing the human networks on MEA with a microfluidic chip that allows the targeting of seizure-inducing and anti-epilepsy drugs to specific networks [142]. Neurotoxicology is one obvious field of application and MEA data parameters such as firing rates, burst rates, burst properties and synchronization provide apt readouts for toxicological analysis of human neuronal networks [94,149,174,175]. The effects of ischemic stroke were also studied in a hiPSC-derived neuronal model of MEA, and it was found that hypoxic conditions (10% air, 90% N_2_) reduce firing rates and disrupt the functional connectivity of human neuronal networks [176]. Recovery of activity (or lack thereof) depended on the duration of the hypoxia. It is evident that hPSC-derived neuronal models of 2D MEA can model various pathological conditions that affect neuronal network activity.

hiPSCs carry the genetic background of their donor, which enables the studying of hereditary neurodevelopmental disorders with 2D cultures of patient hiPSC-derived neurons on MEA. MEA studies on hiPSC-derived neuronal models of neurodevelopmental disorders are listed in Table 1. The table contains studies where the indicated mutations originated directly from the donor and studies where a mutation occurring in human patients was introduced into a hiPSC line. Specific deletions can also be engineered into hiPSC lines: *Cdh13* is associated with both autism and attention-deficit/hyperactivity disorder, and its knockdown in hiPSC-derived GABAergic neurons increases overall inhibition in a network and reduces the duration of synchronous bursts on MEA [135]. Many of the studies on neurodevelopmental disorders studies utilized the Ngn2 and Ascl1/Dlx2 overexpression methods to create glutamatergic and GABAergic neurons that were cultivated either alone [134,177,178,179] or in a mixed culture on MEA [135]. This shows the potential of these differentiation methods in creating hiPSC-derived models of hereditary brain disorders on MEA. Complex neurodegenerative diseases and their individual genetic components have also been studied in hiPSC-derived neuronal models of MEA. These studies are also listed in Table 1. Taken together, these studies indicate that hiPSC-derived 2D neuronal networks on MEA can reveal network dysfunctions that are relevant in neurodevelopmental and neurodegenerative diseases.

## 5. hPSC-Derived 3D Neuronal Cultures and Organoids on MEAs

### 5.1. Properties and Scaffolds of hPSC-Derived 3D Brain Models

Conventional 2D cell culture systems do not mimic several aspects of normal brain development due to the lack of a 3D microenvironment and 3D tissue architecture of the brain [184]. Three-dimensional cell culture models such as organs-on-a-chip, cellular aggregates and tissue explants have been proposed as an alternative to 2D cell culture and animal models as they allow a more feasible study of cell–ECM interactions, cell differentiation, cell-cell connections and electrophysiological network properties [145,185]. In addition, 3D cell culture models mimic fundamental biological processes related to pathogen invasion and drug treatment observed in vivo. The incorporation of synthetic or naturally occurring materials as surface coatings or 3D scaffolds aids in mimicking the 3D in vivo cellular microenvironment and presents physical and biochemical cues to instruct cell fate [185]. Engineered hydrogel matrices proved to be promising scaffolding materials for 3D cell culture models as they closely mimic the natural ECM and allow the testing of biologically active and cell-modulating substances. The mechanical properties of hydrogels can be altered to direct cell differentiation by modification of parameters such as pore size, crosslinking density and topology. Three-dimensional hydrogels that have been studied in combination with neuronal cells include Matrigel, PuraMatrix™, hyaluronic acid, polyethylene (ethylene glycolide)-derivatives, chitosan and nanocellulose [185,186,187]. Non-hydrogel 3D substrates such as the inert Alvetex scaffold [188] have also been successfully used to cultivate hPSC-derived neurons. In summary, hydrogels and other 3D scaffolds have enabled the cultivation of human brain models in a controlled 3D environment that also supports MEA recordings of network activity [70,186,188]. Furthermore, the intrinsic ability of stem cells to assemble into organized clusters of cells within a hydrogel in the presence of suitable exogenous factors paved the way to self-organized tissue organoids [189].

### 5.2. Properties and Differentiation of hPSC-Derived Brain Organoids

Three-dimensional organoid cultures can be generated from a variety of sources such as spheroids, tissue segments or whole organ transplants. In the last decade, the reprogramming process of adult somatic cells into hiPSCs by ectopic expression of pluripotency transcription factors was refined, which led to the successful transformation of these cells into organoids through the activation of signaling pathways involved in the modeling of germ layer formation and the induction of organ primordia [190]. hPSC-derived organoid systems provide a unique opportunity to model human brain development and function due to their ability to self-organize into structures composed of progenitor, neuronal and glial cell types [191,192]. It was shown that, in comparison to corresponding 2D MEA cultures, the firing rates in organoids are drastically higher and the variability in firing rates between replicates is significantly lower [13], even though not all studies report such stark differences between the two model types [183].

Protocols for generating organoids can be classified into either self-patterning (undirected, entire cerebrum) or pre-patterning protocols (directed, region-specific). The serum-free floating culture of embryoid body-like aggregates with quick reaggregation (SFEBq) represents a widely used self-patterning protocol for the generation of 3D brain organoids [193,194]. The protocol includes differentiation of stem cells into neuroepithelial rosettes that show structural similarity to the in vivo cortical neuroepithelium. The optimization of the protocol enabled the production of human-specific outer radial glia (oRG) progenitor cells predominantly found in the human outer subventricular zone (oSVZ) of the neocortex [195]. A modified version of the SFEBq method developed by Lancaster et al. yielded a novel hPSC-based 3D brain model termed the cerebral organoid [6]. Application of growth factors, as in the SFEBq method, was not required in the protocol due to the capability of the organoid to spontaneously acquire various neural cell identities and, hence, to establish multiple regions within a single organoid.

Organoids generated through pre-patterning protocols use small molecules to differentiate the organoid to a certain regional specificity. In the last decade, attempts to model specific brain substructures resulted in the generation of forebrain, midbrain, hippocampal and retinal organoids, including forebrain organoids with full optic vesicles [196,197]. Pre-patterned 3D forebrain organoids of dorsal and ventral telencephalic identity have been fused to allow studies of tissue interactions and the migration of interneurons from the ventral to the dorsal forebrain organoid [198,199,200]. Pre-patterning methods also led to the overcoming of a major hurdle regarding myelination, as adapted pre-patterning protocols were shown to create organoids containing not only neurons and astrocytes but also functional oligodendrocytes [201,202]. Another important step was taken in 2019 when Trujillo et al. reported that in addition to synchronized firing, cortical organoids also displayed delta and gamma waves on MEAs [13]. Taken together, organoids can provide a self-organizing 3D model of the brain (or a brain region) that can display network activity on MEA.

### 5.3. Adaptation of 3D Brain Models to In Vitro MEAs and Vice Versa

Even though hPSC-derived 3D neural models have become more common, practically all commercially available in vitro MEAs are designed for 2D cultures. For example, Ylä-Outinen et al. plated neurons, astrocytes and oligodendrocyte precursors in a hydrogel scaffold on a 2D MEA [186]. Many studies plated pre-differentiated neurospheres or organoids on 2D MEAs from different manufacturers [13,75,150,186,188,203,204,205,206]. The approach has even been expanded to the high-throughput screening of organoids by plating them on 48-well MEAs (Axion Biosystems) [207]. While the approach is valid and can provide a view of the network activity of the culture, the full benefit of having a 3D model is not achieved as the MEA can only provide data from a single 2D plane. It is also possible to slice an organoid for the recordings (Figure 2) [208,209,210] but this is hardly helpful if the goal is to study an intact 3D network from multiple 2D planes. Furthermore, slicing a small organoid and handling the delicate slices (Figure 2) requires specific protocols that include embedding the organoid in an agarose block for slicing and applying sufficient recovery periods before recording. The electrodes of certain arrays do penetrate the sample [69,208,210] (Figure 2), which in itself is a distinct advantage as the dead cell layer on the surface of the sliced sample is overcome, but measurements are still made only from a single 2D plane.

A new approach to simultaneously obtain the electrical activity of 3D cell cultures from several 2D planes is to use a true 3D MEA [211]. As 2D in vivo MEAs were previously used for recording organoids [212], it is conceivable that 3D in vivo MEAs [213] could also be repurposed for organoids and other 3D in vitro models. The approach can provide data that is fully comparable between in vivo and in vitro models, even though the in vitro application of in vivo MEAs may require the building of elaborate micromanipulation setups that enable only end-point recordings [212]. To our knowledge, Soscia et al. published the first work of a successful recording of human neurons in 3D culture with an in vitro MEA where electrodes are dispersed in 3D space instead of a single 2D plane [70]. A single well of the three-well MEA chip had 80 platinum black electrodes (Ø 50 μm) in 10 flexible polyimide shanks that were organized in to four rows with two or three shanks per row. The shank pitch was ~500–700 µm and the electrode pitch was 75 μm. The authors also created a 3D electrode map to better understand the exact topography of their measurements in the culture. The chip was made compatible with a commercially available 256-channel headstage (Multi Channel Systems). Commercially available hiPSC-derived glutamatergic and GABAergic neurons, as well as astrocytes, were suspended in an ECM-collagen gel solution that was allowed to polymerize on the MEA. After 38 days, the mean firing rates (MUA) were from 0.46 to 0.77 Hz and the burst rates were from 3.6 to 4 per min. The networks displayed synchronous spikes even though synchronous bursts were not reported. The activity at this timepoint was, therefore, similar to equally aged 2D cultures from commercial hiPSC-derived neurons [16], but it would be interesting to see if the 3D culture and 3D MEA [70] would be able to show more complex activity at later time points. In 2021, Shin et al. reported an alternative 3D MEA setup where the electrode shanks could be lowered into the sample from above using a micromanipulator [214]. The setup contained 18 silicone shanks with four 20 × 20 μm platinum electrodes in each, and with 85 μm pitch. The shanks were organized in three rows (pitch 500 μm) and six columns (pitch 360 μm). The setup was shown to work with a compartmentalized 3D culture of rat primary neurons in collagen, which displayed synchronized bursts throughout the culture after 14 days. The setup was also tested with a hiPSC-derived spinal cord organoid, which was reported to display array-wide connectivity in EAP firing. Together, these works represent an important advancement of in vitro MEA technology for organoids and hydrogel 3D cultures [70,214].

### 5.4. Modeling Neuronal Development and Physiology with Organoids on MEAs

Brain organoids are considered a model for the early–mid stages of embryonic development. In cortical organoids, both excitatory and inhibitory neurons can display mature function [199,215] and hyaluronan in the ECM appears to be essential to the development of functional inhibitory synapses in organoids on MEA [216]. Moreover, electrophysiology and calcium imaging analyses revealed that functional neuronal properties and synaptic transmission show a trend towards maturation as organoids age [217]. Furthermore, by using MEA, it has been shown that neurons in organoids do not always show spontaneous synchronous network activity, or even firing, but they start to show these features only after several weeks or months in culture, indicating their progressive maturation [212,218,219]. These observations are in line with a study on organotypic slices of human fetal cortex showing that at gestational week (GW) 23, no action potentials were detected in patch-clamp analyses, whereas at GW26, neurons in the deeper layers were able to fire action potentials and exhibit spontaneous synaptic transmission [220].

A 2019 work by Trujillo et al. tried to shed light on the electrophysiological properties of cortical organoids and their possible parallels with human brain development [13]. The authors performed weekly MEA recordings and showed that over the course of 10 months, cortical organoids exhibited a consistent increase in electrical activity, with mean firing rates (MUA) reaching ~17 Hz and burst rates ~15 per min. In detail, cortical organoids started to exhibit highly synchronous and stereotypical network activity at 2 months, which transitioned into 2–3 Hz rhythmic activity by 4–6 months. An increase in spatiotemporal variability of the network activity coincided with the development of inhibitory neuronal populations in the organoid. At 6 months, the oscillatory activity showed cross-frequency coupling between delta and gamma oscillations, a signature of functional neuronal network communication [221]. In order to compare the activity in organoids to an in vivo situation, Trujillo et al. compared a dataset of EEG features from preterm infants to analogous features from the organoid LFP recordings [13]. The authors noticed similarities in development between the two datasets, and a regression model based on the preterm infant EEG features was able to predict the developmental trajectory of LFP activity in the organoids with ~0.6 correlation from week 25 onwards. This suggests that the development of activity in organoids and in the fetal human brain share similarities determined by genetically programmed developmental timelines. The coupling of neural oscillations and the developmental trajectory of electrophysiological activity provided some of the strongest evidence that these organoids can model the complex network activity of the developing human brain.

The maturation of organoids has also been shown when they have been engrafted into the mouse brain [73,222]. Mansour et al. lowered a MEA into the organoid graft and showed that the engrafted organoid exhibited EAPs (SUA) in different sites [73]. Cross-correlation analysis showed a level of synchrony in the EAP firing, suggesting formation of functional neuronal circuits. An increase in the number of electrically active sites and in the number of active neurons in each site suggested progressive maturation of the organoid from 50 to 155 days post-implantation. Moreover, the neurons were able to respond to environmental stimuli, since the firing rates of the neurons gradually increased after removal of isoflurane anesthesia and sharply decreased with its reintroduction [73], similar to a human brain under propofol anesthesia [100]. After the removal of anesthesia, the firing rates varied significantly between neurons but could increase to more than 15 Hz, similar to observations from awake human brains [24,101]. Staining of synaptic markers and optogenetic stimulation of the organoid also suggested the development of functional synaptic connections between the organoid graft and the host brain [73]. The work by Mansour et al. suggests that the maturation of electrophysiological activity also occurs in engrafted organoids, but further analysis will be needed to assess whether the in vivo environment enhances functional maturation of organoids compared to an in vitro environment.

While organoids can be grafted into the brains of mice, certain elements from the in vivo human brain have also been brought into organoids. For example, healthy human CSF was shown to promote the maturation of 3D neural aggregates in only three days, as the CSF-treated aggregates showed suppression of neural stem cell proliferation and an increase in the expression of mature neuronal, glial and synaptic markers [223]. Interestingly, the treated aggregates also displayed significant increases in mean firing rates, burst rates and synchrony on MEA, which was very similar to acute CSF effects in human ex vivo cortical slices [114]. Primary human microglia from mid-gestation aborted fetuses were also grafted into cerebral organoids where they were shown to reduce synapse counts through active pruning of excitatory synapses [224]. The microglia-incorporated organoids displayed a significant increase in synchronous activity on MEA, suggesting that microglial activity contributes to the development of neuronal network function. Neuronal networks in organoids can also respond to physiologically relevant sensory stimuli as shown by whole-brain organoids that contain photo-sensitive neurons [212]. When the organoids were subjected to 530 nm (green) light, the firing rates on MEA reduced significantly. Taken together, MEA recordings of human brain organoids can provide a view into the development of human neuronal networks and into the various physiological factors and cell types that affect the development, maintenance and maturation of network activity.

### 5.5. Modeling Neuronal Pathology with Organoids on MEAs

While brain organoids have already been used to model various brain pathologies from Zika virus infection [225] to obesity [226] and epileptiform activity [15], quite little has been undertaken to track pathological changes in the neuronal network activity of organoids or other 3D brain models using MEA. However, the neurodevelopmental effects of the opioid-replacement drug methadone, for example, were shown to include disruption of neuronal growth and a dose-dependent (transient or permanent) reduction in firing in a cortical organoid model of MEA [227]. On a similar note, lithium salts used as mood stabilizers were shown to cause increased neuronal activity in 3D neural aggregates on MEA, and high concentrations were shown to elicit epileptiform activity [228]. hiPSC-derived cerebral organoids have been used to model certain psychiatric disorders and neurodegenerative diseases, and the studies are listed in Table 2. Even though there are some recent studies utilizing organoids and MEAs in studying brain disorders, they could be more widely applied to model human neuronal network pathology.

### 5.6. Overcoming Limitations of Organoids and Other 3D Brain Models

While brain organoids and other 3D models have solved essential problems, especially regarding the 3D microenvironment, they still have certain limitations. For example, cortical gyrification does not occur spontaneously in brain organoids [191], but requires deletion of the phosphatase and tensin homolog (*Pten)* gene [229] or the use of an engineered microenvironment to create controlled compression [230]. Additionally, the cortical structure is otherwise challenging, as while organoids can develop rudimentary architecture and markers of cortical layers, they still lack the fine architecture of all six layers of the cortex. However, slicing forebrain organoids to 500 μm sections every four weeks was shown to promote the organization of layer-specific markers to resemble the proper layer structure when the sliced organoids were cultivated for more than 100 days [209,210]. Furthermore, a four-shank multi-electrode laminar probe recorded spontaneous firing of EAPs (SUA) and synchronous bursts, showing network activity across long distances both vertically and horizontally in the layer-like structures [209]. An engineered 3D microenvironment might also be able to create an artificial cortical structure; for example, Frega et al. showed that rodent hippocampal neurons can be plated into 5–8 interconnected layers on MEA using glass microbeads as the 3D scaffold [231]. Interestingly, the microbead 3D culture also increased the duration and reduced the counts of synchronized bursts in comparison to 2D culture, indicating increased network excitability.

Three-dimensional models were also found to lack complex interregional neuronal circuitry [191] but certain recent developments may help to overcome this limitation. For example, creating somal and neuritic compartments to hydrogel models [214] and directing neurite growth using gel-embedded nanofibers [232] can help to define neuronal circuitry. Furthermore, fusing pre-patterned organoids (representing different brain regions) in a controlled fashion may also create pre-defined complex circuitry [15,198,199,200].

Because diffusion allows oxygen and nutrients to penetrate less than 1 mm into the tissue, cell viability in deep parts of 3D models can be a problem and organoids tend to develop a necrotic core [191]. The above-mentioned repetitive organoid slicing is one option for allowing oxygen and nutrients to reach the whole culture and preventing necrosis [209]. Similar to classic organotypic cultures, the slices can also be maintained on membranes in air–liquid interfaces, which removes the need for repeating the slicing [208,210]. The membrane-grown slices can be transferred to MEA where they display firing and synchronized bursts. Another option for overcoming the low oxygen and nutrient penetration could be vasculature, and hiPSC-derived epithelial cells have been shown to form vessels into brain organoids [233,234,235]. The vascularized organoids were not reported to display accumulation of necrotic cells [233], supposedly because the medium can reach deep inside the organoid.

Similar to 2D models, low or slow maturation can also be a limitation in hPSC-derived 3D models and organoids. This is evident from processes such as oligodendrocyte production and proper cortical layer organization where stable results can require more than 100 days [201,209]. Still, it is conceivable that the lentiviral overexpression of specific transcription factors, which is used for speeding up and targeting maturation in 2D models [130,131,133], may also be adapted for 3D models. Adaptation of dual-SMAD inhibition to 3D neural aggregates was reported to yield neuronal networks with synchronous bursts and oligodendrocytes in less than 60 days [203]. Considering that it takes more than 70 days to develop oligodendrocyte precursor cells in the human embryo [236], and up to 180 days to develop synaptic activity [220], the protocol represents a significant advancement in 3D differentiation methods [203]. It is also noteworthy that the development of network activity in organoids can recapitulate human embryonic brain development in matching timescales, which can also be seen as a major advantage regarding the relevance of the model [13]. In summary, organoid models currently do not recapitulate the functionality of the adult human brain completely. However, novel technologies, such as vascularization [233,234,235,237] and various bioengineering methodologies [130,131,133,208,209,214,232], are under intensive development and hopefully, in the future, can overcome the current limitations.

## 6. Future Directions

While organoids and other 3D models can provide a more complex model of the human brain, hPSC-derived 2D neural cultures on MEAs will continue to provide relevant information on the function of human neuronal networks in the future. This is shown by the large number of recent publications utilizing the approach to gain insights into pathological network function [7,134,135,143,176,179,180]. However, while it is true that 2D models have considerably less detectable neurons per electrode than corresponding 3D models and the in vivo brain, it would be beneficial to use signal-to-noise ratio-enhancing methods combined with spike sorting to discriminate SUA from 2D models. This would be essential for 1) comparing neuronal firing rates between the human brain and its hPSC-derived models and for 2) determining whether hPSC-derived neurons can be identified as excitatory or inhibitory according to their spike shape and bursting properties [21,101,134,135]. It would indeed be interesting to find means to verify such results in human ex vivo brain samples [110]. Another analysis that can hopefully be extended to 2D cultures in the future is the study of LFP data and neural oscillations. If LFPs are considered as inputs to a specific region [24,34,35], it might be possible to study whether exciting an “emitting” part of the culture would result in specific oscillations in a “receiving” population using MEA-incorporated microfluidic devices [142,159]. This could help us understand network dynamics, e.g., in different types of epilepsy [84,85,108].

Despite the lack of complexity, or even due to the simplicity of the 2D models, they are useful for interrogation of, for example, the function and significance of glia on neuronal network functionality. It would be fascinating to combine microfluidic devices [169] to human neurons and oligodendrocytes [201,202,203] on MEA to study how oligodendrocyte damage or mutations affect axonal signal conduction. Considering that there are ways to compartmentalize hydrogels [214], it might also be possible to study these effects in 3D. The significance of astrocytes to network activity on MEA has been shown [94,173], but future studies are needed to dissect the exact molecular mechanisms by which astrocytes promote network synchronization and how they participate in pathological processes [129]. It is encouraging that the effects of human primary microglia have already been studied in organoids on MEA [224], but considering the poor availability of primary microglia, it would be important to extend the MEA studies to hPSC-derived microglia [117,235,238]. Furthermore, considering the role of neural inflammation in neurodegenerative diseases and infections such as COVID-19 [239,240], it would be essential to study how microglial activation affects network activity in a human context [91].

The connection of brain vasculature and neuronal network function is another aspect that will hopefully be a topic for future studies. Engraftment of vascularized brain organoids to animal models has shown that the in vitro-created blood vessels can connect to preexisting blood vessels of the host, showing the functionality of the vasculature obtained in vitro [73,234]. Mansour et al. recorded the MEA activity of grafted, vascularized organoids but did not study the effects of vascularization on network activity per se [73]. Cakir et al. already analyzed the neuronal activity of vascularized and non-vascularized organoids by whole-cell patch-clamp recordings and showed that the vascularized organoids showed more neuronal activity [237]. The results suggest that the presence of a functional vasculature in organoids plays a role in neuronal survival or maturation, highlighting the need to analyze its impact on neuronal network function using MEA. It is also tempting to imagine a MEA-incorporated vascularized brain model where stimulating the neuronal network activity could lead to a hemodynamic response, or a model where drugs infused through artificial vessels would penetrate through an analogue of the blood–brain barrier and subsequently affect neuronal network activity.

Vasculature also ties the brain to other organs of the body, and it will be essential in the future to study brain models in conjunction with models of multiple organs. The first generation of these body-on-a-chip-models already exists [241], and the incorporation of MEAs into the models can provide important information on the electrical activity of not only the brain-part but also other tissues such as the heart [242], retina [243] and pancreatic beta cells [244]. Furthermore, connecting peripheral neurons to CNS neurons in a body-on-a-chip is an interesting option [245], and the function of the human neuromuscular junction was recently modeled in a microfluidic device on MEA [246].

Even though MEAs have emerged as a vital tool for recording neuronal network activity in hPSC-derived brain models [13], the true 3D MEAs for analyzing 3D cultures are not yet commercially available [70,214]. Furthermore, the creators of these MEAs emphasized that it will be beneficial to increase the electrode density to obtain parameters such as signal conduction velocity and greater coverage of the 3D neuronal network [70,214]. Sufficiently small electrode sizes will also help to better capture SUA [70]. It would also be vital to expand the technology to high-throughput screening in order to gain full benefit for drug discovery [207]. Technological developments have recently also been made in in vivo microelectrode technology; Neuralink corporation aims to use a surgical robot for cortical implantation of wire-connected microelectrodes, which are part of a 1024-channel BMI that promises to allow spinal-cord injury patients wireless use of computers and other devices [247]. It would be interesting to see if this concept and other high-bandwidth and wireless devices [248] could bring additional information on neuronal activity in the human brain.

To conclude, hPSC-derived 2D neural cultures on MEA have established themselves as models of basic network function, although they could describe neuronal circuits in more detail and complexity if combined with microfluidic or micropatterning methods. Organoids and other 3D models need certain fine-tuning in cultivation methods to reach their full potential in representing fine brain architecture and the complexity of in vivo neuronal networks. The 3D models would also benefit from advances in MEA technology that will hopefully be widely available soon. Together, these advances can bring about a new generation of models that can describe the human brain in unprecedented detail and from which neuronal network function can be followed over time in 3D.

## Figures and Tables

**Table 1:** hiPSC-derived genetic 2D neuronal models of neurodevelopmental disorders and neurodegenerative diseases on MEA.

Reference	Modeled disorder	Affected Gene	Neuron Type	Phenotype on MEA
[179]	Koolen-de Vries syndrome	*Kansl1*	Glutamatergic	Mean firing rate ↓ Synchronized bursts ↓
[178]	Mitochondrial encephalomyopathy, lactic acidosis, and stroke-like episodes	*Mt-tl1* (m.3243A > G)	Glutamatergic	Mean firing rate ↓ Synchronized bursts ↓
[177]	Kleefstra syndrome	*Ehmt1*	Glutamatergic	Synchronized bursts ↓ Synchronized burst duration ↑
[180]	Neonatal epileptic encephalopathy	*Kcnq2*	Glutamatergic	Bursts ↑ Spikes per burst ↑
[134]	*STXBP1* encephalopathy	*Stxbp1*	GABAergic	Mean firing rate ↓ Bursts ↓
[181]	Epileptic encephalopathy with intractable seizures	*Scn2a* (L1342P)	Glutamatergic	Mean firing rate ↑ Bursts ↑ Burst duration ↓ Spike frequency in burst ↑ Synchrony ↑
[182]	Amyotrophic lateral sclerosis (ALS)	*Sod1*, *C9orf72* (hexanucleotide repeat expansion)	Motor neurons	Mean firing rate ↑
[143]	ALS	*Tardbp* (Q331K)	Motor neurons	Synchronized bursts ↓ Synchronized burst duration ↑ Signal propagation velocity ↑
[143]	Parkinson’s disease (PD)	*Snca* (A53T)	Dopaminergic	Mean firing rate ↓ Synchronized bursts ↑ Synchronized burst duration ↓
[183]	Alzheimer’s disease (AD)	*Psen1* (M146V), *App* (APP^Swe^)	Glutamatergic (90–95%) and GABAergic (5–10 %)	Mean firing rate ↑ Synchronized bursts ↑

**Table 2:** hiPSC-derived genetic brain organoid models of psychiatric disorders and neurodegenerative diseases on MEA.

Reference	Modeled Disorder	Affected Gene	Organoid Type	Phenotype on MEA
[218]	Bipolar disorder	Multiple	Cerebral	KCl response ↓
[205]	Schizophrenia	Multiple	Cerebral	KCl response ↓
[183]	Alzheimer’s disease (AD)	*Psen1* (M146V), *App* (APP^Swe^)	Cerebral	Mean firing rate ↑ Synchronized bursts ↑
[210]	Amyotrophic lateral sclerosis (ALS) and Frontotemporal dementia (FTD)	*C9orf72* (hexanucleotide repeat expansion)	Cerebral (grown as slice)	No change

## Data Availability

Data are available from the authors upon reasonable request.
